# Imaging Flow Cytometry and Confocal Immunofluorescence Microscopy of Virus-Host Cell Interactions

**DOI:** 10.3389/fcimb.2021.749039

**Published:** 2021-10-12

**Authors:** Ryley D. McClelland, Tyce N. Culp, David J. Marchant

**Affiliations:** Department of Medical Microbiology and Immunology, Li Ka Shing Institute of Virology, Katz Center for Health Research, University of Alberta, Edmonton, AB, Canada

**Keywords:** virus, host, imaging, flow cytometry, confocal microscopy

## Abstract

Viruses are diverse pathogens that use host factors to enter cells and cause disease. Imaging the entry and replication phases of viruses and their interactions with host factors is key to fully understanding viral infections. This review will discuss how confocal microscopy and imaging flow cytometry are used to investigate virus entry and replication mechanisms in fixed and live cells. Quantification of viral images and the use of cryo-electron microscopy to gather structural information of viruses is also explored. Using imaging to understand how viruses replicate and interact with host factors, we gain insight into cellular processes and identify novel targets to develop antiviral therapeutics and vaccines.

## Introduction

Imaging host and pathogen together adds greater dimension to the study of their interactions. This is particularly true for the entry phase of the viral replication cycle. That is, the point at which the virus binds to its cell surface receptor and delivers its nucleocapsid contents into the cytoplasm of the host cell. In a productive viral infection, entry is followed by replication of progeny copies of the viral genome and viral proteins, assembly of these constituents, budding, and release of virus progeny from the infected cell.

**Figure 1:**
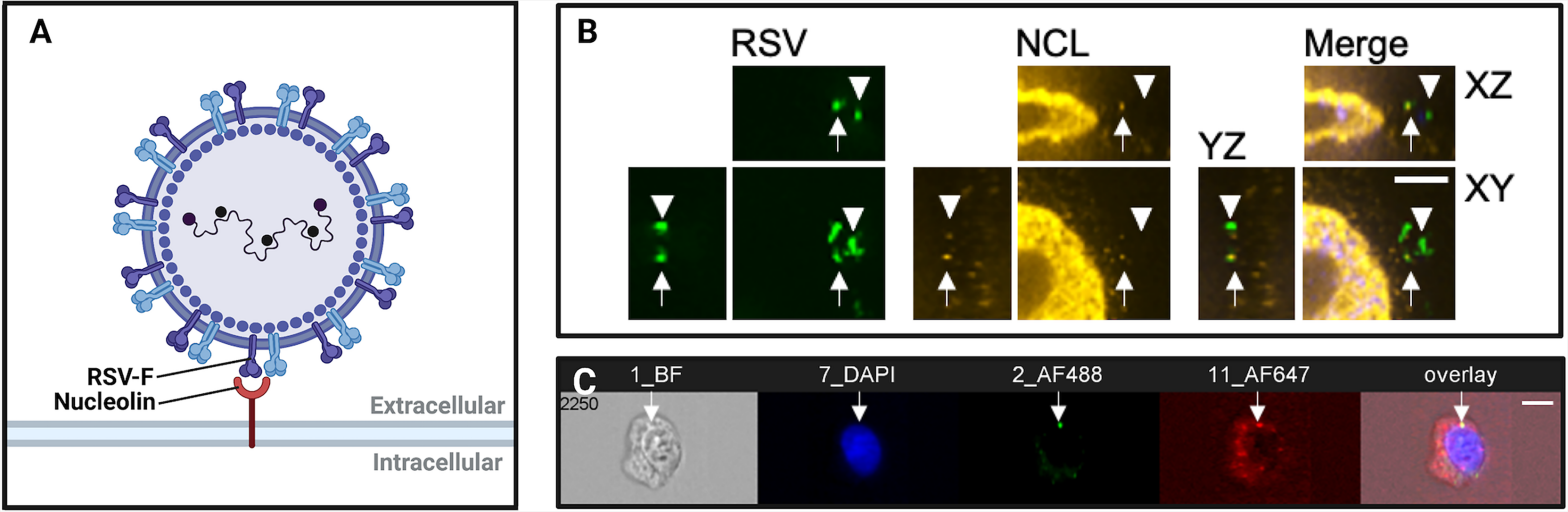
Imaging virus-receptor interactions using confocal microscopy and imaging flow cytometry. **(A)** Schematic representation of respiratory syncytial virus (RSV) binding to nucleolin, an RSV co-receptor, imaged in **(B, C)**. **(B)** Confocal immunofluorescence microscopy of bronchial cells, showing RSV (green), nucleolin (red). Arrows show RSV particles localized to nucleolin, demonstrating typical nucleolin “patching” during RSV infection. Arrowheads denote RSV virions that are not bound to a receptor, and do not co-localize with nucleolin. Scale bar = 6mm. **(C)** Imaging flow cytometry (Imagestream) of RSV and nucleolin. Shown is a representative image out of approximately fifty-thousand recorded events per sample. The overlap of green (RSV) and red (nucleolin) channels was quantified post-acquisition by a user-specified algorithm that determined thresholding and then mask overlap of virus-receptor overlap events. We have found that these ‘overlap events’ detected by imaging flow cytometry corresponded to RSV glycoprotein binding to nucleolin as determined by co-immunoprecipitations, anti-receptor antibody infection neutralization assays, and affinity analysis of recombinant RSV-F glycoprotein and nucleolin.

Imaging virus entry is important because it has helped to identify new virus receptors. It can be used as a tool to provide evidence that a virus is binding to a particular protein or sugar on the cell surface (**Figure 1**). This is important when ascribing the virus entry receptor role to a host cell molecule. Entry receptor binding by viruses is a necessary and crucial step in their replication cycle so it is also a target for the development of antiviral therapeutics.

To image viruses during entry the size of virus particles restricts the ways that they can be imaged. Many viral particles have a diameter that is smaller than the wavelength of visible light, making it impossible to view them through a conventional phase contrast microscope. To directly view viral particles, it is necessary to image by transmission electron microscopy (TEM). However, sample preparation for TEM is laborious. Fluorescence microscopy with imaging flow cytometers and confocal microscopes is typically a more practical and faster method of visualizing viral infections. Labelling of virus particles is indirect in these scenarios, as the virion must be stained by an antibody conjugated to a fluorophore or by staining the viral envelope with lipid dyes. To label viruses directly, viral proteins are tagged with fluorophores such as green fluorescent protein (GFP) by cloning the GFP sequence into the reading frame of a viral protein. These viral labelling methods have been adapted for use in confocal microscopy and imaging flow cytometry. Here, we describe the use of these techniques to study viral entry and replication. Their utility in monitoring viruses in live cells, the importance of image quantification, and the growing popularity of cryogenic electron microscopy (cryo-EM) is also discussed.

## Confocal Imaging

Conventional light microscopy produces significant distortion at resolutions on the scale of micrometers. The advent of confocal microscopy has allowed us to study viruses where they are, and when, in the replication cycle by rejecting out-of-focus light through excitation and emission pinholes to increase optical resolution (Elliot, 2020). Notably, the resolution and spatial information provided by confocal microscopy has aided the characterization of viral entry and pathogenesis mechanisms (Helenius et al., 2001; Coyne and Bergelson, 2006; Coyne et al., 2007; Marchant et al., 2010; Krzyzaniak et al., 2013; Griffiths et al., 2020). The application of fluorescent labelling techniques to confocal imaging has enhanced the specificity and sensitivity of imaging over conventional microscopy, necessary to study the host interactions of viruses. However, imaging must be used in conjunction with conventional biochemical assays for protein and gene expression to support the results of cellular virology studies. In the absence of these additional methods of experimentation, the possibility of analyzing imaging artifacts remains high when imaging is used alone.

Starting with virus entry into the host cell, below we discuss how confocal imaging has been helpful in the study of virology.

### Imaging Virus Entry

The amount of information one may extract from an imaging experiment of virus entry depends on the virus and the infectivity of its virion particles. The relative infectivity of a virus, or the ratio of infective to defective viral particles, can vary between viruses. Infectivity is also influenced by experimental factors such as the host cell line or the method used to purify viral stocks (Bilawchuk et al., 2017). It is easier to image viruses with high infectivity to particle ratios, such as respiratory syncytial virus (RSV) and poxvirus. That is because these viruses typically require only 3-5 viral particles to infect a cell, meaning it is easier to infer that a productive infection will occur from an image of 5 viral particles, compared to viruses with low infectivity ratios (Klasse, 2015). A virus with a low infectivity ratio, like human immunodeficiency virus (HIV) for example, can require up to 10^7^ particles to infect just one cell. This makes it very difficult to determine whether one is imaging infectious or abortive events if viewing just a few dozen virus particles.

There can be multiple pathways into the host cell that do not lead to infection (Schmitz et al., 2004; Mercer and Helenius, 2008; Marchant et al., 2009; Krzyzaniak et al., 2013; Witte et al., 2018). The Helenius group has utilized imaging to discover the basis of virus entry by endocytosis and similar mechanisms to mediate entry by viruses as diverse as poxviruses and pneumoviruses (Mercer and Helenius, 2008; Krzyzaniak et al., 2013). ARF6 mediated endocytosis has been shown to inhibit productive entry by poxvirus and enteroviruses (Mercer and Helenius, 2008; Marchant et al., 2009). The imaging of these viruses during entry and in the presence of overexpressed ARF6 had the seeming ability to reduce infection. Images show trapped virus in an ARF6-mediated endocytosis compartment that was separate from productive entry routes (Marchant et al., 2009).

Recently, we used confocal imaging as a critical part of the discovery of the RSV receptor, insulin-like growth factor-1 receptor (IGF1R) and its coreceptor, nucleolin (NCL) (Griffiths et al., 2020). IGF1R was perhaps a more obvious candidate than NCL as a member of this receptor family because epithelial growth factor receptor (EGFR) had been suggested to be a candidate receptor previously (Krzyzaniak et al., 2013; Currier et al., 2016). Here we used fixed confocal immunofluorescence of monolayers and differentiated airway epithelial cultures to observe RSV particles interacting with IGF1R and then its coreceptor, NCL. An instance of this interaction is shown in **Figures 1A, B**. The triggering of RSV entry fusion after interaction with NCL was imaged by live cell imaging, that we discuss later in this review.

The application of confocal imaging has been critical to confirm surface NCL expression and describe its roles in host-pathogen interactions as a receptor and attachment factor (Semenkovich et al., 1990; Hovanessian et al., 2000; Nisole et al., 2002; Hovanessian et al., 2010). NCL is translocated to the cell surface by an unconventional mechanism that is independent of the ER-Golgi peptide secretion complex (Hovanessian et al., 2000). In HeLa, MT-4, CEM, and PBMC cells, imaging of NCL revealed organized clustering of NCL at the plasma membrane within glycosphingolipid- and cholesterol-rich lipid rafts (Hovanessian et al., 2000; Nisole et al., 2002). Confocal imaging of murine tumor cells was used to confirm cell-surface expression of NCL *in vivo* (Christian et al., 2003). In Tayyari et al. (2011), we used confocal immunofluorescence imaging to support NCL as a receptor for RSV. Using a Leica SP2 laser scanning confocal microscope, we found that RSV and NCL colocalized at the apical surface of polarized 1HAEo- cells (Tayyari et al., 2011). RSV infectivity and colocalization with NCL were reduced in the presence of NCL-blocking antibodies, which suggested that NCL is a receptor for RSV (Tayyari et al., 2011). In another study, confocal microscopy was used to track the cellular entry and interaction of Ebola virus particles with lipid rafts, demonstrating the importance of lipid raft integrity for Ebola virus pathogenesis (Jin et al., 2020).

Confocal imaging has been a useful supplementary tool in cellular virology studies which investigate entry mechanisms and inhibitory molecules to address the current pandemic attributed to severe acute respiratory syndrome-coronavirus-2 (SARS-CoV-2). In Yang et al. (2020), the binding dynamics between the angiotensin-converting enzyme 2 (ACE2) receptor and the S1 subunit of the SARS-CoV-2 spike protein were assessed with a confocal microscope coupled to an atomic-force microscope. In this example, confocal microscopy validated the expression of ACE2-eGFP at the cell surface and guided atomic force microscopy to assess ACE2-S1 molecular interactions under various conditions (Yang et al., 2020). In another study, confocal imaging detected GFP-tagged SARS-CoV-2 virus-like particles to confirm the cellular-entry and utility of a safer biosafety level 2 (BSL-2) model of SARS-CoV-2 infection (Plescia et al., 2021).

In summary, confocal imaging helps to confirm in an image whether one sees colocalization of a receptor candidate with virus particles. One must keep in mind the infectivity ratio of the virus in the cell type that is being imaged. Of course, imaging must be done as a supplemental experiment and doesn’t replace traditional plaque and infectivity experiments. All of these experiments together are required to support a candidate virus discovery.

### Virus Replication and Budding

Confocal imaging has helped elucidate viral mechanisms of replication and egress from the host cell for a wide array of viruses (Mercer and Helenius, 2008; Rincheval et al., 2017; Evans et al., 2020). Both host and viral-based fluorescent fusion proteins and immunofluorescence techniques have been used in conjunction with confocal microscopy to study viral replication and budding (Liu et al., 2020). The direct labelling of viral proteins with conjugated fluorophores has been extensively applied to image and study the intracellular behavior of viruses such as HIV, vaccinia virus, and SARS-CoV-2 (McDonald et al., 2002; Jouvenet et al., 2008; Mercer and Helenius, 2008; Plescia et al., 2021).

Copper-catalyzed click chemistry has been used to label nascent nucleic acids of viral genomes undergoing replication in addition to proteins and micro-RNAs interacting with the viral genome (Kolb et al., 2001). Developments have expanded the application of click chemistry to label lipids, amino acids, and sugars (Moses and Moorhouse, 2007; Izquierdo and Delgado, 2018). These advancements have been of particular utility to the field of virology, as the cellular interactions between virus and host during infection often involve dynamic interplay between lipids, sugars, proteins, and nucleic acids.

The viral budding process enables viral particles to exit host cells and acquire a host-derived membrane (Chazal and Gerlier, 2003; Rossman and Lamb, 2010). Viral budding is coupled to virion assembly. In a productive virus infection cycle, *de novo* synthesis of the viral genome and proteins initiates the replication and assembly of progeny virions. For RSV, replication complexes were found to associate with internal cellular membranes enriched in lipid rafts (McDonald et al., 2004). Rincheval et al. (2017) found that RSV inclusion bodies (IBs), or replication complexes, are organized structures in which newly-synthesized viral RNAs are concentrated with the M2-1 viral protein in IB subcompartments, termed IB-associated granules (IBAGs). In this study, confocal imaging and fluorescence *in situ* hybridization (FISH) was used to detect host or viral RNA and stain RSV-infected cells (Rincheval et al., 2017).

Confocal immunofluorescence imaging is useful for studies investigating the subcellular location and components of viral replication. The Coyne group have studied the molecular biology of RNA virus replication extensively. In Evans (2020), the Coyne group demonstrated that BPI fold-containing family B member 3 (BPIFB3), a regulator of autophagy, is required for efficient replication of zika virus and dengue virus in human endothelial cells. Levels of zika virus and dengue virus replication after BPIFB3-silencing were quantified by staining viral double-stranded RNA (dsRNA) replication intermediates in infected cells and quantifying the associated fluorescence with confocal microscopy (Evans et al., 2020).

In virology, confocal imaging helps to characterize the mechanisms of viral replication and budding by visualizing findings within the three-dimensional context of the host cell. With proper labelling techniques, one can discern where and what viruses are interacting with in the host cell during replication and assembly of progeny virions. Confocal imaging is a useful tool in the study of virology, helping us gain a better understanding of the cellular processes involved in the viral replication cycle to identify novel antiviral and vaccine drug targets.

## Live Cell Imaging

Live-cell imaging can provide valuable insight when studying the dynamic entry process viruses have evolved to invade host cells. When combined with fluorescent microscopy and supravital dyes, live-cell imaging greatly enhances our understanding of the dynamic and multi-step nature of viral infections. Importantly, dyes that do not interfere with viral infectivity or cellular functions must be used (Sun et al., 2013).

Used in tracking infection pathways and replication cycles, lipophilic dyes help to provide the spatiotemporal information pertinent to understanding viral infection processes. Hydrophobic-lipophilic interactions permit membrane permeability, facilitating the incorporation of lipophilic dyes into the host membrane-derived envelopes of viruses (Liu et al., 2020). Commonly used fluorescent lipophilic dyes, such as the DiD, DiL, DiO, and DiR dyes, are often used to label hydrophobic targets like membranes or viral envelopes. At high concentrations, the fluorescence of lipophilic dyes tends to self-quench due to close-proximity constraints (Klymchenko et al., 2012; Wagh et al., 2012). When viral membranes fuse with the membranes of host cells and endosomes, the dyes diffuse across the membranes, resulting in dequenching and an increase in emitted fluorescence indicating the membrane fusion event (Loyter et al., 1988; Lowy et al., 1990). Fluorescence dequenching from endosome-viral membrane fusion has helped characterize the infection pathway of influenza viruses and dissect it into a three-stage pre-fusion process by tracking single DiD labelled viruses in real-time in living cells (Lakadamyali et al., 2003). The self-quenching lipid analogue R18 has also been useful to study Influenza A virus (IAV) envelope fusion with host cell membranes. Lowy et al. (1990) were able to continuously visualize IAV membrane fusion, quantifying the transfer of R18 from IAV envelopes to infected erythrocyte membranes.

Our group found that IGF1R recruited NCL, the coreceptor for RSV fusion, to the cell surface where the virus awaited entry (Griffiths et al., 2020). To do this we used a DeltaVision OMX structured illumination microscope. The super resolution function of this microscope was not used, though it’s higher resolution camera was useful to capture these virus-receptor interactions with RSV particles stained with DiD. Target cells expressed a NCL construct that expressed GFP in frame with the NCL protein. This allowed us to image RSV fusion and entry upon clustering with surface trafficked NCL-GFP by fluorescence in real-time.

Prior to the discovery of the RSV entry receptor, IGF1R (Griffiths et al., 2020), early stages of RSV infection and entry into human bronchial epithelial cells were characterized using lipophilic fluorochromes R18 and DiOC18 (San-Juan-Vergara et al., 2012). Here, the self-quenching of lipophilic dyes at high concentration was taken advantage of to identify membrane fusion events between RSV and host cells, as quenching allows for single-labeled virions to be detected. Similarly, by monitoring the presence or absence of lipophilic dye diffusion, it was identified that complete HIV-1 fusion occurs with endosomes but not host plasma membranes (Miyauchi et al., 2009). This aided in the characterization of the entry mechanism of HIV-1 *via* endocytosis and endosome fusion. Recently, the conventional use of lipophilic dyes with viruses has influenced the development of quantum dots (QDs), nanosized semiconductors, for use in virology (Zhang et al., 2020). Similar to long-chain lipophilic dyes, viral lipid membranes have been modified and conjugated with QDs, taking advantage of the high optical quality of QDs for use in live cell imaging.

## Imaging Flow Cytometry

**Figure 2:**
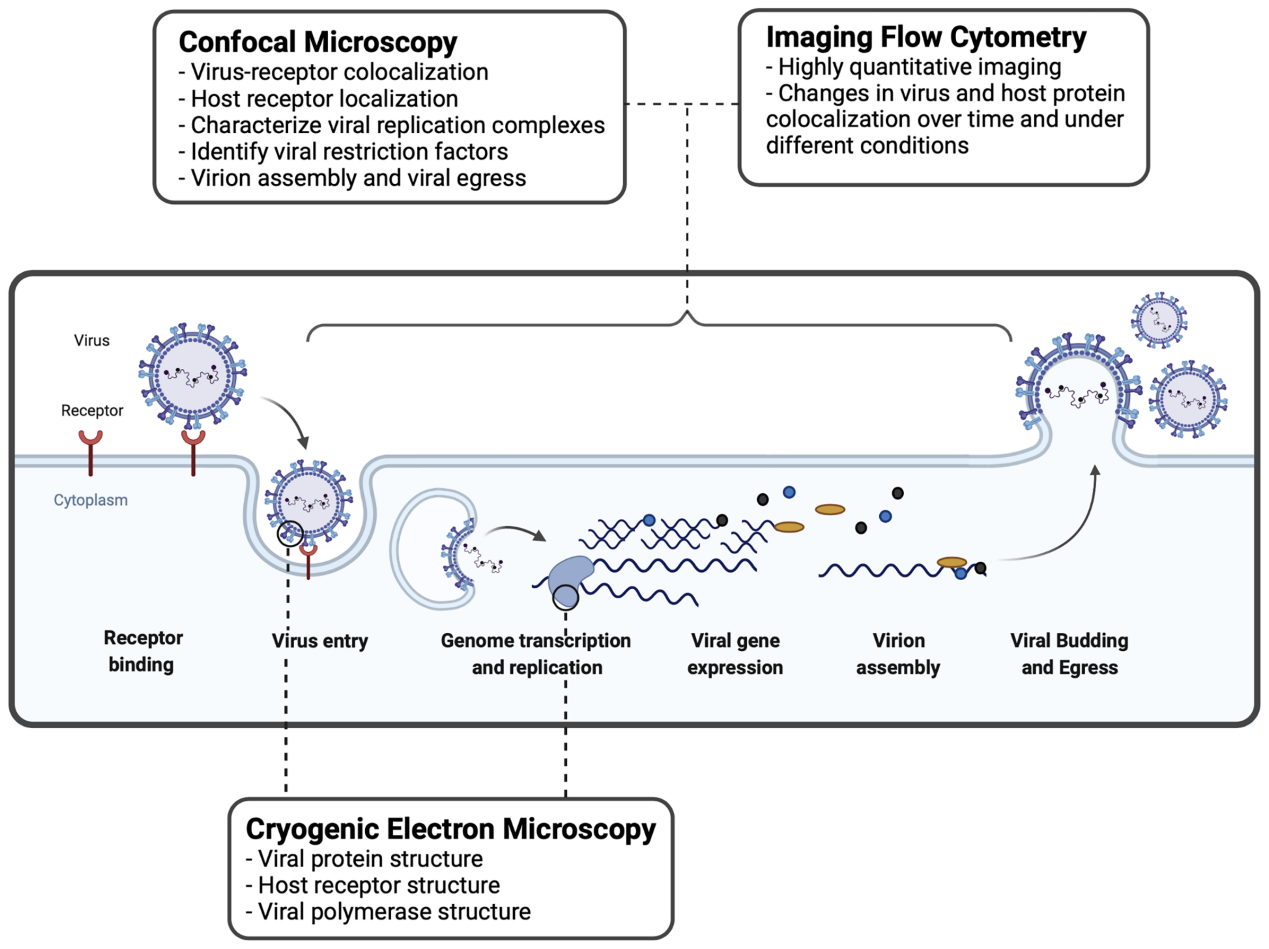
Imaging techniques used to study virus-host interactions during the viral replication cycle. A basic viral replication cycle for RSV, an enveloped -ssRNA virus, is shown. All viruses follow the general principles of cellular entry, genomic replication, virion assembly, and virion release during a productive infectious cycle. Confocal microscopy, imaging flow cytometry, and cryogenic electron microscopy are three common imaging techniques used within the study of virology to assess interactions between virus and host cells.

Merging the principles of fluorescence microscopy and flow cytometry, imaging flow cytometry (IFC) enables high-throughput, concurrent analysis of cell morphology and multi-channel fluorescence imaging of single cells (**Figure 1C**). With the emergence of the ImageStream-100 (Amnis corp.) in 2005, IFC has since become a valuable tool to study interactions between host and virus (Basiji et al., 2007). Over the past decade, advancements to IFC technologies have increased spatial resolution while conserving the high-throughput quantitation of conventional flow cytometers. Similar to confocal microscopy, IFC generates data regarding fluorescence of labelled structures. Unlike confocal microscopy, IFC captures an image of every cell and then quantifies the fluorescence of thousands of individual cells within a single run to generate statistically robust datasets. In virology, however, IFC is often performed in parallel with confocal microscopy to generate observations that are statistically robust and backed by 3-dimensional imaging (Bilawchuk et al., 2017; Griffiths et al., 2020). The high-throughput and highly-quantitative nature of IFC has been helpful to characterize subtle host-pathogen interactions and limit the analysis of imaging artifacts (**Figure 2**).

The main limitation of IFC is that combining high-resolution, multi-spectral imaging with high sample throughput produces complex datasets with extremely large file sizes. Images produced by IFC are rich in information compared to conventional flow cytometry, but the computational and back-end data processing requirements have prevented IFC from becoming widely accepted ( Han et al., 2016). Software such as IDEAS by Amnis has made it easier to process and analyze IFC datasets, but file sizes have remained cumbersome (Basiji et al., 2007; Barteneva et al., 2012; Han et al., 2016). To gain broader acceptance and increase practical functionality, imaging technologies for flow cytometry should prioritize the development of multimodality, functional flexibility, and the incorporation of fluorescence-activated cell sorting in addition to methods for efficient data handling and transport (Han et al., 2016).

IFC is utilized by virologists to track the intracellular distribution and localization of viral proteins or nucleic acids, within the morphological context of a single cell (**Figure 2**). IFC has been useful to study interactions between viruses and host immune factors. Below, we describe how IFC has been applied in the field of virology to study virus entry and replication.

### Imaging Flow Cytometry to Study Virus Entry

IFC has enabled virologists to analyze subtle interactions between virus particles and host receptors at the cell surface (**Figures 1C**, **2**). These discoveries have contributed to the elucidation of novel receptors and host factors contributing to viral infection. IFC has played a valuable role in the discovery of drug targets aiding the development of antivirals and vaccines.

Recently, Hudak et al. (2021) used IFC to assess the contribution of syndecans, a family of transmembrane proteoglycans, to the cellular entry of SARS-CoV-2. Using GFP-tagged syndecan constructs and immunolabeled SARS-CoV-2 viral proteins, data gathered by IFC revealed the colocalization and increased cellular uptake of the S1 subunit of the SARS-CoV-2 spike protein with syndecan-4 (Hudak et al., 2021). In another example, IFC was used to image ‘patching’ of the RSV coreceptor NCL at the cell surface (Bilawchuk et al., 2017; Griffiths et al., 2020). Shown in **Figure 1C**, the colocalization of fluorescent NCL constructs and RSV-GFP particles within lipid rafts on the cell surface were quantified, demonstrating focal colocalization that increased following infection (Bilawchuk et al., 2017; Griffiths et al., 2020). IFC is a powerful tool to elucidate novel virus entry receptors. Syndecan-4 and cell surface NCL are two receptors that were characterized by IFC and found to mediate the viral entry mechanisms of SARS-CoV-2 and RSV, respectively. As such, these surface proteins identified by IFC are novel targets for antiviral drugs.

Viral pathogens have evolved entry mechanisms that take advantage of essential cell surface receptors to invade our cells. Likewise, our innate cellular defenses have evolved unique mechanisms to counter infection. IFC is an excellent tool to study host factors suppressing viral entry as it can be used to track protein translocation and colocalization. One example is the suppression of endosome-mediated entry by interferon-inducible short isoform of human nuclear receptor coactivator 7 (NCOA7). Doyle et al. (2018) demonstrated that NCOA7 inhibits influenza A virus membrane fusion by activating vacuolar ATPase to induce vesicle acidification and lysosomal protease activity. Importantly, membrane fusion, haemagglutinin acidification, and vRNP import assays were performed with IFC, demonstrating the functional flexibility and practical range of IFC in studying viral entry (Doyle et al., 2018).

### Imaging Flow Cytometry to Study Virus Replication

IFC is a proven tool to characterize viral replication. ImageStream analysis of labeled viral replication intermediates and proteins has enhanced our understanding of the host-pathogen interactions during virus replication. Moreover, the data gathered by IFC analysis has been important to aid the development of effective therapeutics for viral infections such as hepatitis C virus (HCV), human cytomegalovirus (HCMV), and IAV. The lack of a vaccine and limited therapeutic options for HCV is due, in part, to the lack of a small animal model of HCV infection (Dorner et al., 2013). In an interesting example using IFC to quantify viral replication, Dorner et al. (2013) developed a humanized transgenic mouse model of HCV infection stably expressing CD81 and occludin, to enable HCV entry. To validate the HCV mouse model, *in situ* HCV replication was quantified using ImageStream X analysis and a transgenic fluorescent reporter activated by HCV NS3-4a cleavage, where HCV RNA replication directly correlates to the level of reporter activation (Dorner et al., 2013).

The statistically-relevant throughput and spatial information provided by IFC has been useful to enhance the development of IAV vaccines. Recent advances in computational biology have enabled researchers to model virus-host interactions during IAV replication (Laske et al., 2019). In a study by Laske et al. (2019) IFC analysis was applied to validate mathematical models of IAV replication to develop novel vaccine producer cell lines. Specifically, the nuclear import kinetics of labelled IAV nucleoproteins were quantified to assay permissibility of IAV infection in genetically engineered A549 cell lines (Laske et al., 2019). In another example, IFC was used to optimize IAV production in tissue culture by analyzing the localization of viral nucleoprotein within nuclear regions of interest, and the colocalization of IAV protein fluorescence with DAPI signal (Frensing et al., 2016).

In a recent example demonstrating the use of IFC to image host-pathogen interactions during HCMV replication, Businger et al. (2019) utilized IFC to characterize the unique HCMV mechanism to usurping restriction factors of viral replication in macrophages. Immunostaining of intracellular HCMV immediate-early 1 and 2 (IE1/2) antigen and antiviral restriction factor sterile alpha motif and histidine-aspartate domain-containing protein 1 (SAMHD1) enabled IFC analysis of SAMHD1 expression in HCMV-infected cells (Businger et al., 2019). In this study, SAMHD1 was able to suppress HCMV replication in macrophages, but IFC analysis showed that HCMV has evolved countermeasures to usurp SAMHD1 suppression by interfering with SAMHD1 expression (Businger et al., 2019).

## Image Quantification

The quantification of viral images is necessary to fully exploit the potential of imaging systems and remove experimental bias (Hamilton, 2009). The quantification and statistical analysis of viral images allow valid inferences to be made on virus entry and replication mechanisms. In virology, imaging observations are broadly quantified by measuring the relative fluorescent intensity of molecular probes.

It is important to ensure that the integrity of quantitative images are not compromised by poor experimental design, image acquisition, or analysis (Jonkman et al., 2020). It is necessary to image high-quality samples and create pipelines for image acquisition and analysis to ensure reproducibility and generate imaging data that is meaningful and quantifiable (Hamilton, 2009; Jonkman et al., 2020). Methods to ensure quality image acquisition and quantitation have been reviewed in detail elsewhere (Hamilton, 2009; Kelley and Paschal, 2019; Shihan et al., 2021).

Regardless of the imaging modality used, researchers must analyze and interpret increasingly large volumes of data as imaging technology advances. Software such as IDEAS™, Volocity™, and ImageJ™ exist to streamline the imaging quantification pipeline and decrease the burden of image analysis. In virology, Volocity image analysis software has been used extensively to process and quantify confocal images of virus-host interactions, such as the downregulation of peroxisomes by zika virus or the trafficking of HCV core proteins during virion assembly (Counihan et al., 2011; Wong et al., 2019). ImageJ macros have been developed to automate the quantification of fluorescence microscopy images and help quantify virus infected cells (Handala et al., 2019). Researchers using IFC to image virus-host interactions are burdened by large datasets that are difficult to quantify without IFC-specific software, such as the Imagestream IDEAS image analysis software package. The IDEAS software can quantify multiple parameters of single cells within IFC datasets, including the relative fluorescence intensity, colocalization of probes, and cell shape among others (Basiji et al., 2007).

To quantify viral entry and replication with confocal imaging, the levels of labelled molecules in a cell are measured relative to other molecular probes or within specific regions of interest (Marchant et al., 2010; Krzyzaniak et al., 2013). By measuring the fluorescent intensity of two probes, labelling virus particles and host receptors, statistical analysis using Pearson’s correlation coefficient is a reliable method to quantify virus-receptor colocalization (Dunn et al., 2011). It is important to note that correlation between two probes can be under-represented if Pearson’s correlation coefficient is measured across an entire field of cells instead of a specific, marked, region of interest (Dunn et al., 2011). Imaging can also be used to quantify the localization of viruses or viral proteins in cells to assess virion internalization in the context of viral entry or immune clearance (Smirnov et al., 2015; Griffiths et al., 2020). Similar quantification philosophies are applied when imaging viral infections in live cells. When normalized to cell area, the quantified fluorescence of activated reporters, antibodies, and dyes have been vital to investigate viral entry and replication (Marchant et al., 2010; Dorner et al., 2013; Griffiths et al., 2020).

The same principles used to quantify fluorescence by confocal microscopy can be applied to imaging flow cytometry, with the added statistical power provided by imaging thousands of cells. High-throughput IFC allows the researchers to quantify abstract and concrete cellular characteristics, such as cell morphology or fluorescence intensity within regions of interest (Hamilton, 2009). The changes to cell morphology induced by a viral infection can be quantified by IFC, utilizing the brightfield and side-scatter channels of imaging cytometers (Young and Morrison, 2018; Zamora and Aguilar, 2018). With the large sample sizes analyzed by IFC, the kinetics of viral infections can be assessed by quantifying the location of viral proteins or virion particles relative to cellular structures (Laske et al., 2019; Yaakov et al., 2019). For example, the nuclear import of IAV genomes was quantified using IFC by co-staining cells with DAPI and anti-IAV ribonucleoprotein antibodies and measuring the increase in nuclear fluorescence (Laske et al., 2019). Other parameters quantified by IFC include cellular granularity, or side-scatter, which can indicate the accumulation of progeny virions intracellularly or at the cell surface (Yaakov et al., 2019). This is exemplified in Yaakov et al. (2019), where increased side-scatter intensity was first observed by IFC 2 hours after mimivirus infection, indicating intracellular progeny virus production. Developments in IFC sample throughput, image acquisition, and data processing will benefit the quantification of host-virus interactions during viral infection cycles.

## Cryo-EM Elucidation of Protein Structure

Cryogenic electron microscopy (cryo-EM) is an increasingly used, powerful technique capable of high resolution determination of macromolecule structure. Prior to imaging, the protein sample is flash-frozen and contained within an amorphous layer of ice that offers protection from both beam-induced radiation damage and the high vacuum of the microscope (Bai et al., 2015). Flash-freezing makes crystallization unnecessary for preservation, conserving native, functional protein conformations, conferring potential to provide incredible virological insight (Renaud et al., 2018).

Despite the method of flash-freezing molecules being developed by Dubochet et al. as early as 1981, (Dubochet et al., 1982) only recently have significant technological advances led to extensive expansion in the use of cryo-EM. The first advancement contributing to this rapid expansion is the development of direct electron detectors, which allow for greater signal-to-noise ratios, owing to improved detection quantum efficiency (DQE) when compared with past detectors (Bai et al., 2015). Additionally, these detectors permit blurring compensation during imaging (Campbell et al., 2012; Li et al., 2013). The second central improvement is the increase in processing power and advancement of software algorithms, increasing user-friendliness, imaging complexity, and efficiency (Punjani et al., 2017).

The recent advances in cryo-EM, coupled with its ability to elucidate structures and conformations of complex and dynamic proteins without crystallization in their native, fully functional states, solidify cryo-EM as a powerful tool in structural virology (Goldsmith and Miller, 2009). Many of the advances made in cryo-EM have been accomplished using viruses as model systems. In fact, the use of cryo-EM for characterization of any biological specimens was first demonstrated with structural analysis of adenovirus and bacteriophages, alongside several other viruses (Adrian et al., 1984). In 2010, the first viral atomic structure produced by cryo-EM was also of adenovirus, where the entire human adenovirus virion structure was reported at 3.6 Å resolution (Liu et al., 2010).

Cryo-EM characterization of viral structures has provided great insight into viral replication, entry/infection, assembly, and the functionality of viral components.

Cryo-EM analysis of the RSV RNA polymerase has suggested potential new targets for future RSV polymerase inhibitors (Cao et al., 2020). Promising protein targets likely cannot be mutated by the virus in order to escape inhibition as a mutation could be functionally deleterious due to proximity to the polymerase’s active site. Similarly, in cryo-EM studies of the RNA polymerase of influenza A virus, potential target sites identified for the development of antivirals include polymerase dimerization sites and cRNA promoter-binding sites (Fan et al., 2019). Discoveries such as these based on cryo-EM imaging have been used to inform potential new vaccines and antibody designs against other viruses, including HIV, group B coxsackieviruses, and SARS-CoV-2 (Lee et al., 2016; Wang et al., 2021; Xu et al., 2021). Recently, structural information gathered *via* cryo-EM has characterized helicase-promoted backtracking activity by the RNA polymerase of SARS-CoV-2 (Malone et al., 2021). Backtracking is central to proofreading and thus may aid in antiviral resistance.

## Discussion

Visualizing and quantifying virus-host interactions is crucial to understanding viral infections. Viral entry and replication are critical stages to study in any effective investigation. Confocal microscopy and imaging flow cytometry are practical, efficient techniques for imaging these processes. Live cell imaging, coupled with lipophilic dyes to label virus and host, makes tracking dynamic viral infections possible. Statistical quantification of viral images and cryo-EM structural information also aid in the understanding of viral infections. Viral imaging critically expands our understanding of host-pathogen interactions and helps identify novel targets conferring potential to develop antiviral therapeutics and vaccines. However, it must be considered that viruses are dynamic, intracellular, and among the smallest known pathogens. This complexity means that careful sample fixing, preparation, and staining are required to image viruses by confocal microscopy and imaging flow cytometry. This manipulation of course inserts the possibility of artifacts in images. Once we can effectively image nascent and unadulterated virus particles in live cells, we will truly reveal the nature of virus entry and replication.

## Author Contributions

RM conceived and wrote the manuscript. TC conceived and wrote the manuscript. DM conceived and wrote the manuscript. All authors contributed to the article and approved the submitted version.

## Funding

Canadian Institutes of Health Research grant number: RN382934-418735.

## Conflict of Interest

The authors declare that the research was conducted in the absence of any commercial or financial relationships that could be construed as a potential conflict of interest.

## Publisher’s Note

All claims expressed in this article are solely those of the authors and do not necessarily represent those of their affiliated organizations, or those of the publisher, the editors and the reviewers. Any product that may be evaluated in this article, or claim that may be made by its manufacturer, is not guaranteed or endorsed by the publisher.
